# Does reducing smartphone use impact physical activity?

**DOI:** 10.1371/journal.pone.0311248

**Published:** 2024-10-11

**Authors:** Annaëlle Le Steunf, Ewen Page, Yannick Guillodo, Alain Saraux

**Affiliations:** Service de rhumatologie, CHU Brest, INSERM 1227, Université de Bretagne Occidentale, LabEx IGO, Brest, France; University of Montenegro: Univerzitet Crne Gore, MONTENEGRO

## Abstract

**Introduction:**

A sedentary lifestyle and low levels of physical activity among the population are major public health challenges due to numerous associated health risks. These behaviors are influenced by a number of environmental and cultural factors, such as screen addiction.

**Objective:**

To study the impact of reducing smartphone use by one hour per day for one week on physical activity.

**Method:**

We conducted a descriptive epidemiological study that included 490 individuals. Participants were recruited by means of a media campaign called “Let’s put down our smartphones challenge”, which was conducted in Brest, France. The questionnaires were widely publicized, and candidates from all over France were able to participate. Data reflecting physical activity were collected before and after the challenge, one week apart, through an online questionnaire.

**Results:**

Among the 490 participants, 126 (25.7%) succeeded in the challenge and reduced the time spent on their smartphones by more than 60 minutes. Among those 126 participants, 112 individuals (89%) increased their physical activity. On average, participants who succeeded in the challenge reduced their smartphone use by 110.57 minutes (±53.66) (*p*< 0.001) and increased their number of steps by 841 (±14710) (*p* = 0.02). The proportion of patient reaching an increase of both 500 and 100 daily steps was statistically significant in the group reaching a reduction of smartphone use of 60 minutes versus the remainder (p = 0.016 and 0.018, respectively), but not for a cut off at 1000.

**Conclusion:**

A reduction in the time spent using smartphones results in an increase in the average number of daily steps. Limiting the leisure time spent on screens is a potential strategy for addressing physical inactivity and a sedentary lifestyle.

## Introduction

A sedentary lifestyle and low levels of physical activity (PA) among the population are major public health challenges due to numerous associated health risks [1]. Sedentary behavior is defined as any waking activity with an energy expenditure less than or equal to 1.5 METs [2]. Sedentary behavior includes time spent sitting or lying down or standing in a static position that requires low levels of energy expenditure. The meaning of physical inactivity differs from that of a sedentary lifestyle. Physical inactivity is characterized by a low level of moderate-to-vigorous PA. The World Health Organization (WHO) published global recommendations regarding PA and a sedentary lifestyle for all sections of the population [3].

Currently, we have many studies with high levels of evidence that highlight health dangers associated with physical inactivity and sedentary behavior [4]. The main risks directly associated with these behaviors are an increase in the mortality rate and the prevalence of chronic diseases. [4–7]. In 2008, the WHO released an alarming report indicating that insufficient PA is the fourth leading risk factor for premature mortality. Approximately 3.2 million deaths worldwide each year are attributed to insufficient PA. These health risks also have socioeconomic impacts, and by 2030, “high-income countries […] will account for 70% of health-care expenditure on treating illness resulting from physical inactivity”, according to the latest report by the WHO [8]. However, the numerous recommendations from learned societies do not seem to be considered as people are becoming increasingly sedentary from a younger age. However, sedentary behavior is even more dangerous when it is combined with a low level of PA: the deleterious effects of sedentary behavior and a low level of PA can potentiate each other.

Globally, sedentary behavior affects all countries. The average time spent on sedentary activities in many countries is estimated to be between 8.5 hours and 10.5 hours per day. This sedentary time is directly linked to an increase in all-cause mortality [9]. In France, according to the 2022 report published by The French Agency for Food, Environmental and Occupational Health Safety (ANSES) [10], 95% of adults do not reach the duration and frequency thresholds of PA recommended by the WHO.

The direct consequence of this new addiction to screens is “chair addiction” and therefore physical inactivity and sedentary behavior. The idea for the “Let’s put down our smartphones” challenge in Brest came from this observation. This prevention and awareness raising campaign is based on a German study conducted in 2022 [11]. The findings of this study showed that abstaining from using a smartphone or reducing its use by one hour per day for one week led to a healthier lifestyle and increased PA. This study also showed that the effects were greater and more stable over a four-month period in the reduction group than in the abstinence group.

The “Let’s put down our smartphones” challenge invited the population from Brest to reduce their screen time by an hour per day and over a seven-day period. This extra time provided the opportunity to engage in leisure activities that did not include the use of a smartphone. The purpose of this initiative was to raise public awareness of the health problems associated with the excessive use of smartphones. A website was created by the Communication Agency Lumy (www.posons-nos-smartphones.fr) to motivate and reassure participants.

The challenge was covered by different groups, including the local media, UCPS BO (Université Citoyenne de Prévention en Santé de Bretagne Occidentale), and the Brest football club (le Stade Brestois), and was supported by local sportsmen. Moreover, several businesses were approached about participating in the challenge in an attempt to raise awareness to the maximum number of people.

The objective of this work was to evaluate the impact of reducing smartphone use by one hour per day for one week on PA.

## Materials and methods

### Type of study

This quantitative and descriptive epidemiological study was conducted using declarative data through an online questionnaire before and after a media campaign.

### The population being studied

Participants were recruited by means of a media campaign for the “Let’s put down our smartphones” challenge. The volunteers were asked to complete an online questionnaire at the start of the challenge and another one after completing the challenge. The challenge was to reduce the screen time by one hour per day for 7 days from the 21^st^ to 27^th^ of November 2022 and to analyze the impact on PA. Volunteers were required to own a smartphone to participate in the challenge. Participation in the questionnaire was anonymous and free and was mainly intended for inhabitants from the Brest region. However, the surrounding towns were also invited to participate.

All individuals registered for the “Let’s put down our smartphones” challenge who had both provided their email address and filled in both questionnaires were included. However, individuals who had not provided their email address or had only completed one questionnaire were excluded. The number of required research participants was not calculated.

### Ethics

As it was an anonymous study on mobile phone data, ethic committee board approval was not needed (confirmed by the Territorial Ethical Reflection Committee, Brest). We obtained from patient their approval for use their online questionnaire filled at the start of the challenge and after completing the challenge from the 21^st^ to 27^th^ November 2022, and to analyze the impact on physical activity.

### Questionnaire (S1 Annex)

The first part of the questionnaire surveyed characteristics of the population taking part in the challenge to determine their sociodemographic profile, including their age, gender, weight, height, marital status, education, residence, parental status and occupation. Then, the average number of steps was reported as well as the average time spent on a smartphone every day. Technical help with obtaining these data was offered within the questionnaire. The second questionnaire contained the same questions apart from the questions about sociodemographic characteristics.

The average number of steps taken daily provided insight into physical activity patterns. The number of steps could be calculated using the accelerometer of a smartphone or connected watch. The average daily screen time could be directly reported using the statistics provided in the phone settings under “Screen Time”. This average provides an estimate of sedentary time.

All participants were invited to provide their email address to merge their answers from the first and second questionnaires.

### Statistics

Numeric variables are expressed as the mean (±SD), and discrete outcomes are expressed as absolute and relative (%) frequencies. Homogeneity of the measure T1 and T2 were evaluated using alpha Cronbach. Continuous outcomes were compared with Kruskal‒Wallis test. Discrete outcomes were compared with those from the chi-squared test or Fisher’s exact test. The alpha risk was set to 5%, and two-tailed tests were used.

Spearman’s correlation was used to assess the linear dependence between reducing smartphone use and increasing average daily steps. Correlations were judged as very strong from 1 to 0.9, strong from 0.9 to 0.7, moderate from 0.7 to 0.5, low from 0.5 to 0.3 and poor from 0.3 to 0. The alpha risk was set to 0.05.

To illustrate with binary variables the effect of the reducing smartphone use and increasing average daily steps, we compared the proportion of patient reaching an increasing of daily steps of 10, 500 and 100 in the group reaching or not a reduction of smartphone use of 60 minutes.

Then, we evaluated the number of patients needed for further studies according to the increasing number of steps using the software Biostatgv (https://biostatgv.sentiweb.fr.

Statistical analysis was performed with both EasymedStat (Paris, France) and SPSS 25.0 (IBM, NY, USA) statistics.

## Results

### Descriptive analysis

The questionnaires were created using the Google Forms platform (S1 Annex), after which all the collected data were entered into a Microsoft Excel spreadsheet. Privacy and confidentiality protections on the survey included anonymous data collection. All questions were broad to avoid any chance of identification of individuals. Informed consent was obtained upon opening the survey. There was no time limit to complete the questionnaire. Completion rate by the 400 respondents was 100%. There was no compensation and no physical risks were associated with the survey.

**Fig 1:**
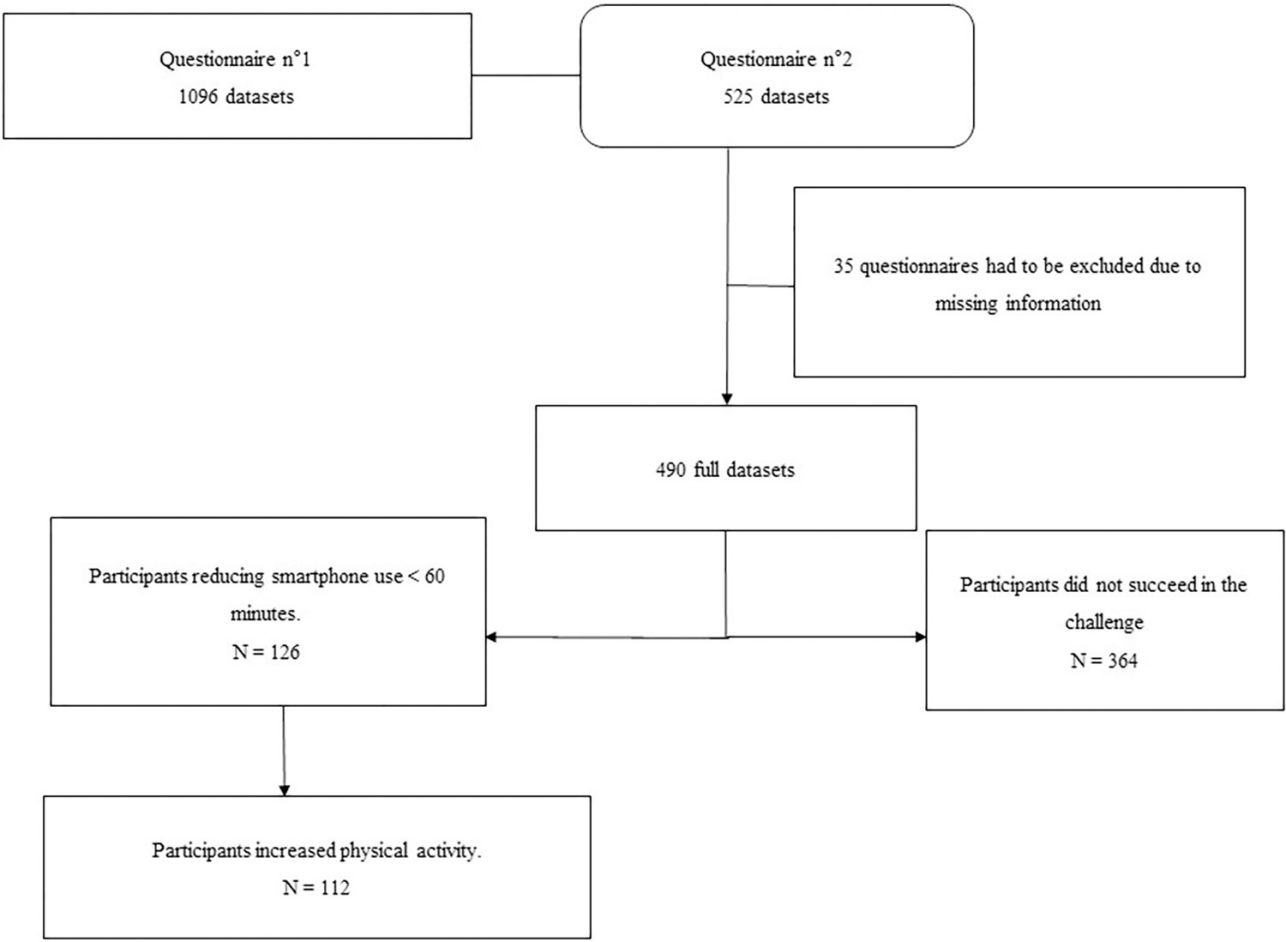
Flow chart.

The first questionnaire was collected between 27 September 2022 and 9 December 2022, and 1096 questionnaires were collected. The data for the second questionnaire were collected between 29 November 2022 and 10 January 2023, and 525 questionnaires were collected. We were able to use 525 datasets for comparison of the results from the first and second questionnaires. Among those datasets, 35 questionnaires were excluded due to missing information. Therefore, the analysis included 490 questionnaires (**Fig 1**).

### Characteristics of the participants

**Fig 2:**
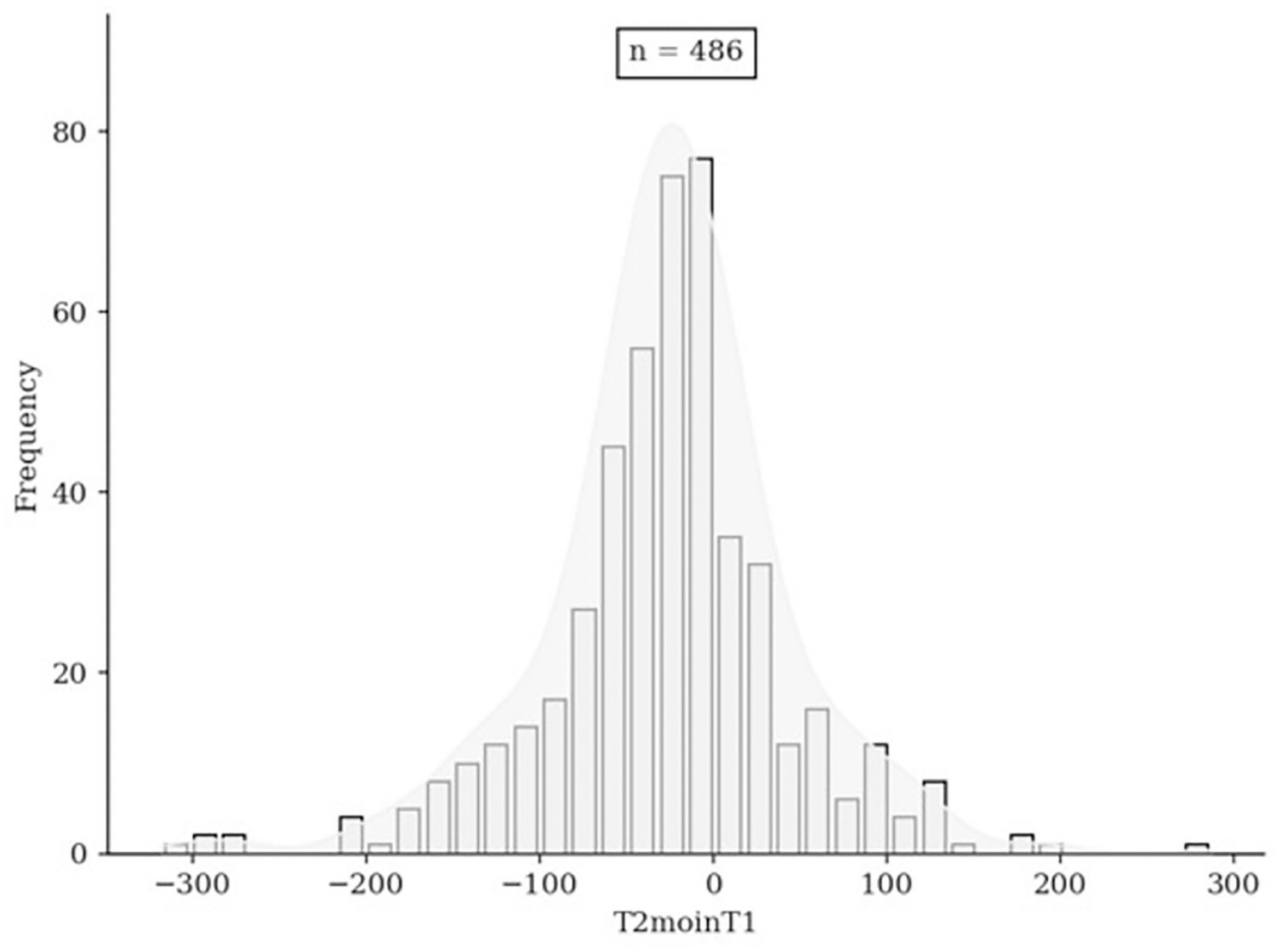
Histogram showing the change in screen time of participants (in minutes) after the challenge.

The main characteristics of the population are shown in **Table 1**. The average age of the participants was 39 years (±13). The sex ratio was 0.4 (M/F). The average BMI was 23.96 kg/m2 (±4.8). On average, individuals used their smartphones for 184.8 minutes (±99.6) per day before the challenge compared to 159.7 minutes (±99.5) after the challenge. The average number of steps before the challenge was 12,190.2 (±19,05), and the average number of steps after the challenge was 12,045.15 (±19,23). Fig 2 shows the change in screen time of participants after the challenge.

**Table 1:** Characteristics of patients who decreased their screen time by more than 60 minutes after the challenge.

Characteristics	decrease <60 min n = 364	decrease ≥60 min n = 126	p value
Females, n (%)	109 (29.95%)	34 (26.98%)	0.606
Age (years), mean (SD)	39.43 (± 13.04)	38.5 (± 12.16)	0.573
BMI, mean (SD)	24.01 (± 4.69)	24.23 (± 5.19)	0.574
***Place of residence***			0.327
• Large city, n (%)	135 (37.09%)	54 (42.86%)	
• Medium size city, n (%)	35 (9.62%)	5 (3.97%)	
• Small size city, n (%)	101 (7.75%)	33 (26.19%)	
• Town, n (%)	62 (17.03%)	23 (18.25%)	
• Village, n (%)	31 (8.52%)	11 (8.73%)	
***Education***			0.549
• More than 3 ***years*** baccalaureate, n (%)	223 (61.26%)	73 (57.94%	
• Between one and three years after baccalaureate, n (%)	100 (27.47%)	35 (27.78%)	
• Baccalaureate level, n (%)	25 (6.87%)	8 (6.35%)	
• Less than baccalaureate, n (%)	14 (3.85%)	10 (7.94%)	
• Other, n (%)	2 (0.54%	0 (0.0%)	
Single or divorced, n (%)	113 (31.04%)	37 (29.37%)	0.81
Screen time (min) before the challenge, mean (SD)	159.7 (± 89.94)	244.41 (± 97.7)	< 0.001
Screen time (min) after the challenge, mean (SD)	167.28 (± 114.13)	133.84 (± 80.3)	0.003
Number of steps before the challenge, mean (SD)	12102 (± 19715) N = 328	11202 (± 16160) N = 115	0.966
Number of steps after the challenge, mean (SD)	11501 (± 19790) N = 332	12849 (± 16538) N = 120	0.104

### Decrease in screen time and increase in physical activity after the challenge

Among the 490 participants, 126 individuals succeeded in the challenge, and they reduced the time spent on their smartphone by more than 60 minutes. Among those 126 participants, 112 increased their PA. On average, participants who succeeded in the challenge reduced their screen time by 110.57 minutes (±53.66) (*p value* < 0.001) and increased their daily PA by 841 steps (±14,71) (*p value* = 0.02). The 364 individuals who failed the challenge had, on average, increased their screen time by 7.6 minutes (±79.8) (*p value* <0.01) and even reduced their level of PA by less than 485 steps (±18,71) (*p value* = 0.02 on a daily average).

### Correlation between the reduction in screen time and increase in the number of steps

The correlation coefficient between ranks (Spearman’s rho) was -0.103 (p value = 0.03). This finding implies that there is a weak but significant correlation between the reduction in screen time and the increase in the number of steps. Therefore, this result seems to show that the more time spent on a smartphone, the greater the decrease in PA.

We also investigated whether there was a correlation between the reduction in time spent on a smartphone and the sociodemographic characteristics of participants in the challenge. No associations were found between the reduction in time spent and sex, age, education, BMI, marital status, or place of residence.

### Number of patients needed for further studies according to the increasing number of steps after a challenge

The proportion of patient reaching an increase of both 500 and 100 daily steps was statistically significant in the group reaching a reduction of smartphone use of 60 minutes versus the remainder (p = 0.016 and 0.018, respectively), but not for a cut off at 1000 (Table 2). So, the number of patients needed for further studies according to the increasing number of steps is very high to demonstrate an increase of 1000 steps (n>2000) but it is low to demonstrate an increase of 500 or 100 steps (n <400) (Table 3).

**Table 2:** Increase in physical activity (100 step, 500 steps or 1000 steps) after the challenge according to decrease in screen time < or ≥60 minutes.

Increase in physical activity after the challenge	decrease <60 min n = 324	decrease ≥60 min n = 112	p value
**Increase in physical activity ≥ 1000**	114 (35.19%)	45 (40.18%)	0.405
**Increase in physical activity ≥ 500**	132 (40.74%)	61 (54.46%)	0.016
**Increase in physical activity ≥ 100**	147 (45.37%)	66 (58.92%)	0.018

**Table 3:** Number of patients needed for further studies evaluating the increase in physical activity according to the number of steps.

Increase in physical activity after the challenge	≥ 1000	≥ 500	≥ 100
Number of subjects needed	2316	362	312

## Discussion

The purpose of this work was to evaluate the one-hour reduction in time spent on a smartphone and its impact on PA. The statistical analysis showed that 89% of participants who succeeded in the challenge were able to increase their PA. The sociodemographic characteristics did not have any significant influence on the reduction in screen time or increase in PA according to multivariate analysis. Individuals who failed to reduce their screen time, or 74% of the sample, even increased their sedentary behavior.

The “let’s put down our smartphones” challenge was a primary preventive action. We encouraged participants to adopt a healthy lifestyle through the www.posons-nos-smartphone.com website. People were encouraged to choose leisure activities such as music, cultural days, manual activities and physical activities. This action helped people to become aware of their daily smartphone use. It has been proven that excessive use of screens for leisure activities is harmful to public health. There are many health risks, and excessive use leads to a sedentary lifestyle.

People have been preferring a sedentary lifestyle for many years. This increase in sedentary behavior is strongly linked to industrialization as well as the improvement of new technologies such as means of transport, television, and the automation of many jobs, often creating a sedentary society.

Although they are different from each other, a sedentary lifestyle and physical inactivity both have adverse effects on health, so much so that the combination of these behaviors can cause serious risks to our health. From the middle of the 20^th^ century [12], dangers associated with a sedentary lifestyle were brought to the fore, and in subsequent years, these risks were clearly defined. People with sedentary behavior have a 20% higher risk of suffering from noncommunicable disease, decreased insulin sensitivity, obesity or developing chronic diseases [13]. A prospective American study from 2016 showed that a one-hour increase in sedentary time was associated with a 12% greater mortality risk. This same study also demonstrated that any effort to reduce sedentary time would be beneficial for our health. Replacing one hour of sedentary time with one hour of light-intensity activity is associated with an 18% lower mortality risk [14].

Regarding cardiovascular diseases, the Journal of the American College of Cardiology states that there is a 48% increased risk of all-cause mortality in those spending more than 4 hours a day in a sitting position. There is a 125% increase in the risk of cardiovascular events in those spending more than 2 hours a day in a sitting position [15]. It is worth mentioning that these risks are increased regardless of other cardiovascular risk factors, such as high blood pressure, tobacco use, overweight status, cholesterol and the level of PA. Other studies agree with these results–an Australian study provides similar results. This study also revealed that each 1-hour increment in sitting position was associated with an 11% and an 18% increased risk of all-cause and cardiovascular event mortality, respectively [16]. Diabetes, one of the main risk factors for cardiovascular disease, is a pathology that can lead to serious long-term complications [17]. According to Grøntved and Hu, sitting for more than two hours per day while watching TV increases the risk of type 2 diabetes by 20% [18].

A sedentary lifestyle is a risk factor for cancer pathogenesis and spread. The National Cancer Institute studied a cohort of 488,720 people and showed that sedentary behavior is associated with the risk of developing colorectal cancer. With regard to sedentary behavior in men, for those who spend more than 9 hours of screen time per day, the risk of colon cancer is increased by 61% compared to that for those who spend less than 3 hours of screen time per day [19]. A prospective cohort study including 40,000 women showed that a sedentary lifestyle can have an impact on endometrial cancer incidence. Indeed, a sedentary lifestyle of more than 5 hours a day is associated with a 66% increased risk of endometrial cancer [20]. It also impact sleep quality [21].

A sedentary lifestyle can also cause musculoskeletal disorders. For example, smartphone overuse can cause neck and shoulder muscle fatigue and pain. This phenomenon was studied by a Korean team who demonstrated that fatigue and pain worsen with longer smartphone use. Regular breaks of at least 20 minutes are necessary to limit the negative impacts of smartphone use in the long term [22].

We reviewed all organs and systems to determine whether adverse consequences were directly or indirectly associated with a sedentary lifestyle. Based on this observation, it is essential to address the factors that increase sedentary time. This objective is proving to be challenging: our study showed that reducing screen time by one hour seemed difficult, as 74% of our cohort did not reach this objective despite a strong incentive.

It is essential to educate children because they are highly likely to engage in sedentary behaviors. Eighty percent of children and teenagers do not reach physical activity level targets in association with increased sedentary behavior. Sedentary behavior particularly affects young girls.

We focused on leisure screen time because it is challenging to reduce work-related screen time. Daily screen time is an indicator of sedentary behavior levels. Smartphones are good examples of leisure screen time because they are widely used to surf on the internet, watch videos, listen to music, and play video games. Smartphone ownership is very high among the population. The smartphone is the most commonly used device in digital leisure activities. Smartphones are the primary devices we use to occupy our free time [23]. Therefore, smartphones were the most relevant devices for calculating screen time use and the number of steps. The data were not declarative but were directly established using smartphone settings.

This type of original study is, to our knowledge, the first to be carried out in France. The study method ensured that the population was adequately represented. However, selection bias is linked to regional characteristics. The Elfe study states that there is a significant regional disparity regarding average screen time in France. The daily screen time among children is indeed lower in Brittany than in Hauts de France (47 minutes vs. 1 hour and 4 minutes)[24]. Despite the large volume of initial data, the challenge was subject to volunteer bias, as nonrespondents to the second questionnaire were not included.

In addition, this study presented limitations inherent to declaration and understanding bias. Despite a pretesting of the questionnaire, some of the questions might have been reworded to enhance the reliability of the results. A study performed in the general population do not have the exactness of a clinical study. Nevertheless, this study confirm the acceptability and the potential use of media to induce behavioral modifications in the general population.

This study revealed a positive correlation between daily reductions in smartphone use and increases in PA and that it is feasible to evaluate the proportion of patients reaching an increase of 100 to 500 steps after a media campaign aimed to decrease screen time of 60 minutes. Our goal now is to conduct a large study aimed to study if it is feasible and relevant to increase the average number of daily steps >500 in the general population.

To conclude, this study showed that reducing the amount of time spent on a smartphone increases the average number of daily steps. Fighting against excessive time spent on screens for leisure activities is an avenue to explore to address physical inactivity and sedentary behavior.

## Supporting information

S1 Annex Questionnaire in French. (DOCX)

S1 Data (XLS)
