# Supplementary material for: Does reducing smartphone use impact physical activity?
Source: PLoS One. 2024 Oct 11;19(10):e0311248. doi: 10.1371/journal.pone.0311248 (PMC11469508; doi:10.1371/journal.pone.0311248)
Supplement: S1 Annex — (DOCX) [file pone.0311248.s001.docx]

Annex 1: Questionnaire in french

Vous venez de vous inscrire à l'action "**posons nos smartphones**" !

En répondant à ce questionnaire simple, vous permettez à 3 étudiantes en médecine à Brest de faire leur thèse de doctorat. L'intérêt de notre étude est de mesurer l'**impact de la diminution du temps d'écran sur la santé**, notamment l'**activité physique** et le **sommeil**.

Par ailleurs, un deuxième questionnaire vous sera adressé, fin Novembre, après la semaine d'action.

Quoi faire ? **regarder le temps d'écran sur son smartphone, enlever 1h de ce temps et ne pas dépasser cette limite pendant une semaine.**

**Comment** **connaître** **mon** **temps** **de** **consommation** **de** **smartphone** :

*Pour* *Apple* :

1. Accédez aux **Réglages** de votre téléphone

2. Appuyez sur **Temps** **d'écran**

3. S'il n'est pas activé, appuyez sur **Activer** "**Temps** **d'écran**"

*Pour* *Android* :

1. Ouvrez les **Paramètres** de votre téléphone

2. Appuyez sur **Bien-être** **numérique** et **contrôle** **parental**

3. Dans la section "Vos outils Bien-être numérique", appuyez sur **Afficher** **vos** **données**

**Pensez à activer le podomètre sur votre smartphone :**

*Pour Apple :*

1. Pour activer la fonctionnalité "Mouvements et forme", allez dans “Réglages”

2. Appuyer sur “Confidentialité”, puis “Mouvements et forme”

3. Validez le bouton “Suivi forme” et activez l’application “Santé”

*Pour Android :*

Suivant la marque de votre téléphone sous Android, une application de suivi de nombre de pas existe. Il peut s'agir de Google fit, Samsung Health ou Huawei Santé par exemple. Pour l'activer, il faudra sûrement vous rendre les réglages de votre téléphone puis dans Confidentialité.

Sexe*

Homme

Femme

Année de naissance ? *

Votre réponse

Poids (en Kg) *

Votre réponse

Taille (en cm)*

Votre réponse

Vous habitez dans un(e)*

Village : < 2000 habitants

Bourg : >2000 et <5000 habitants

Petite ville : >5000 et <20000 habitants

Ville moyenne : >20 000 et <50 000 habitants

Grande ville : >50 000 habitants

Niveau d'instruction*

Inférieur au BAC

BAC

entre 1 an et 3 ans après le BAC

plus de 3 ans après le BAC

Autre :

Statut marital*

Marié(e) / concubinage / PACS

Célibataire / divorcé(e) / veuf(ve)

Autre :

Statut parental : indiquez le nombre d'enfants*

Votre réponse

Profession exercée*

Agriculteur exploitant

Artisan, commerçant ou chef d'entreprise

Cadre et profession intellectuelle supérieure

Profession intermédiaire

Employé

Ouvrier

Retraité

Etudiant

Autre personne sans activité professionnelle

Autre :

Comment vous êtes-vous inscrit à cette action "posons nos smartphones"*

En mode individuel

Dans le cadre de votre entreprise

Autre :

Heure de téléphone et pas avant

Quel est votre temps moyen d'heure passé sur votre smartphone ? (… Heures et …. Minutes)

Quelle est la moyenne du nombre de pas de la semaine dernière ?

Heure de téléphone et pas après

Quel est votre temps moyen d'heure passé sur votre smartphone ? (… Heures et …. Minutes)

Quelle est la moyenne du nombre de pas de la semaine dernière ?
